# Knowledge and practices related to antimicrobial use in fighting cocks – a survey of fighting cock owners in Timor-Leste

**DOI:** 10.3389/fcimb.2025.1569037

**Published:** 2025-09-29

**Authors:** Abrao J. Pereira, Frederico Amaral, Mauricio J. C. Coppo, Kirsten E. Bailey, Shawn Ting, James Gilkerson, Glenn F. Browning, Joanita Bendita D. C. Jong, Jenny-Ann L. M. L. Toribio

**Affiliations:** ^1^ Animal Health Department, Faculty of Agriculture, Universidade Nacional Timor Lorosae, Dili, Timor-Leste; ^2^ National Directorate of Veterinary, Ministry of Agriculture, Livestock, Forestry, and Fisheries, Dili, Timor-Leste; ^3^ The Asia Pacific Centre for Animal Health, Melbourne Veterinary School, The University of Melbourne, Parkville, VIC, Australia; ^4^ Escuela de Medicina Veterinaria, Facultad de Ciencias de la Vida, Universidad Andres Bello, Concepcion, Chile; ^5^ Global and Tropical Health Division, Menzies School of Health Research, Charles Darwin University, Darwin, NT, Australia; ^6^ School of Veterinary Science, Faculty of Science, The University of Sydney, Sydney, NSW, Australia

**Keywords:** fighting cock, antibiotics, antimicrobial resistance, knowledge, practices, Timor-Leste

## Abstract

**Introduction:**

Cock fighting is an industry with a long standing in Southeast Asia and the birds have a high value. However, there is scant published literature on antimicrobial use and knowledge about antimicrobial resistance (AMR) among fighting cock owners in Timor-Leste. This survey assessed knowledge of fighting cock owners about antimicrobials and AMR, and their use of antimicrobials in fighting cocks.

**Method:**

This was a cross-sectional survey conducted on randomly selected owners of fighting cocks in urban areas in Timor-Leste between February and March 2023. Data collection was done using a structured questionnaire administered by face-to-face interviews.

**Results:**

A total of 275 participants were interviewed. Knowledge about antimicrobials and AMR among fighting cock owners in Timor-Leste was poor. Owners in urban areas (aOR = 2.4, 95% CI [1.4-4.1], p-value = 0.002) and those with higher education (aOR = 2.3, 95% CI [1.3-4.2], p-value = 0.007) were more knowledgeable about antimicrobials. The majority used antimicrobials (76.2%, 205/269) in their cocks and the most common antimicrobials used were amoxicillin (77.5%, 159/205) and ampicillin (54.2%, 111/205). The most common reasons for use were for treating fight wounds (85.4%, 175/205), respiratory signs (26.3%, 54/205), and diarrhea (21.0%, 43/205).

**Conclusion:**

This study revealed widespread antimicrobial use by fighting cock owners with low awareness about AMR, which creates a high-risk environment for selection for emergence of AMR. There is a need for a comprehensive intervention that combines regulatory controls, enhanced surveillance, and a targeted campaign to mitigate public health risks in Timor-Leste.

## Introduction

Antimicrobial resistance (AMR) is a growing global concern and is considered among the top 10 global health problems (WHO, 2020). It emerges when microorganisms such as bacteria gain the ability to resist the effects of the drugs that are used to treat infections (Jonas et al., 2017; Nadeem et al., 2020). Globally, deaths associated with AMR are estimated at around 700,000 people annually (Pokharel et al., 2020), with the highest mortalities reported in Africa and Asia (Kariuki et al., 2022). If this problem remains unaddressed, human deaths are projected to reach 10 million annually by 2050, associated with an estimated economic loss of around USD 100 trillion (O’Neill, 2016). These human health impacts will be more pronounced in low-and-middle-income countries (LMICs), mainly in Africa and Asia, which are the key global hotspots for AMR (Ikhimiukor et al., 2022; Murray et al., 2022).

The capacity of existing antimicrobials to effectively treat common bacterial diseases in both humans and animals is limited by AMR. As a consequence, many diseases are becoming more difficult and more expensive to treat (Friedman et al., 2016; WHO, 2020). Many factors have contributed to rising AMR, but a key driving factor is the misuse and overuse of antimicrobials (Dadgostar, 2019; Caneschi et al., 2023). Global usage data indicates that the majority (reported to be 73% in the mid-2010s) of about antimicrobials are administered to animals (Van Boeckel et al., 2019), largely for routine disease prevention and growth promotion in intensive livestock farming systems (Roope et al., 2019; Pokharel et al., 2020), although antimicrobial use for growth promotion has been forbidden in many countries, including those belonging to the European Union (Gonzalez Ronquillo and Angeles Hernandez, 2017), the United States of America (Hoelzer et al., 2017), and major meat producing countries in South America (Da Silva et al., 2023). The global consumption of antimicrobials in animal production is estimated to be between 63,000 - 240,000 tons per year (Jonas et al., 2017). Resistant bacteria in livestock have the potential to spread to humans, particularly in settings where people live in close proximity to their animals and have higher levels of interaction with them, such as in smallholder livestock production systems in low-income countries (Ikhimiukor et al., 2022).

Fighting cocks are central to a long-standing, global animal gambling industry. Cockfighting has been banned in high-income countries on animal welfare grounds, but it is widely practiced in many countries in Latin America, Africa, and Asia (Chakraborty, 2018). However, there are few publications in the peer-reviewed literature reporting the management and health of these birds. For reasons including cultural practice and affordability, fighting cocks are commonly raised and kept by smallholders in many parts of Southeast Asia (Pfeiffer et al., 2013). Interest in ownership of these birds among smallholder farmers is attributed to their higher economic value and social worth (Valeix, 2012). In Timor-Leste, cockfighting is popular and deeply embedded in Timorese culture (De Andrade, 2023), although its exact origin is unknown. Apart from being a source of entertainment for men, with attendance at cock fights culturally restricted to males, it is viewed as a business that involves people from various walks of life (Hutt, 2015; De Andrade, 2023). The breeding of fighting cocks is an important aspect of cockfighting and is an alternative income source for many households (De Andrade, 2023), and the roosters that die in cockfights are commonly consumed by their owners and their families (Wu, 2022).

Cockfighting activities have been linked with disease outbreaks and the spread of zoonotic diseases of public health concern, such as highly pathogenic avian influenza (Pfeiffer et al., 2013; Hassan, 2014). Spread of pathogens occurs as a result of the regular handling and care of the birds by people and the transport of fighting cocks between locations, including, unofficially, between countries (Sims et al., 2005). This potential for zoonotic risk can include AMR. A survey in southern Thailand that collected samples from fighting cocks found evidence of high levels of resistance to penicillins in staphylococci (Fungwithaya et al., 2022). The broader environmental risks are substantial, as waste from the birds, including feces that is probably contaminated with resistant bacteria, is often disposed of directly into the environment, posing a risk to humans and other animals (Wongtawan et al., 2022). Fighting cock activities often occur in an unsanitary environment where infection prevention and control measures are absent (Pacelle, 2020). The lack of such controls at fighting pits exacerbates the risk of both zoonotic transmission and environmental spread of AMR.

Measures to preserve the efficacy of antimicrobials need to be implemented with urgency across medical and veterinary settings. In Timor-Leste, there are ongoing coordinated efforts in response to the risks posed by AMR. A national action plan has been developed to guide collaboration between stakeholders to collectively address AMR (WHO, 2022). Under this, antimicrobial use and systems for monitoring AMR in human and animal health have commenced (Francis et al., 2020). This has provided data about antimicrobial consumption and resistance to inform strategies to address AMR. In the animal health sector, around 229.8 kg (mean, 57.4 kg; s.d., 31.0 kg) of active antimicrobials, mostly tetracyclines, penicillins, and macrolides, were imported into Timor-Leste between 2016 and 2019 (Ting et al., 2021). A survey for resistant *Escherichia coli* in local chickens and fighting cocks found higher resistance to tetracyclines and penicillins than to other classes of antimicrobials (Pereira et al., 2024). While key programs, such as surveillance and regulation of antimicrobial use, are important AMR mitigation strategies (Uchil et al., 2014), understanding how animal owners are using antimicrobials in their animals, and their knowledge about antimicrobials and AMR is crucial to identifying knowledge gaps and practices of animal owners that can be modified by targeted interventions (Subedi et al., 2023). Such studies have been carried out in many countries, including in some LMICs (Emes et al., 2023; Subedi et al., 2023). However, to date, in Timor-Leste, studies to evaluate knowledge and practices have been limited to pig farmers (Ting et al., 2022a) and government animal health workers (Ting et al., 2022b). To our knowledge, no study has been conducted with a focus on fighting cock owners in the Pacific region. Thus, this study aimed to investigate the knowledge of fighting cock owners in Timor-Leste about antimicrobials and AMR, and to describe owner use of antimicrobials in fighting cocks.

## Materials and methods

### Study area

**Figure 1:**
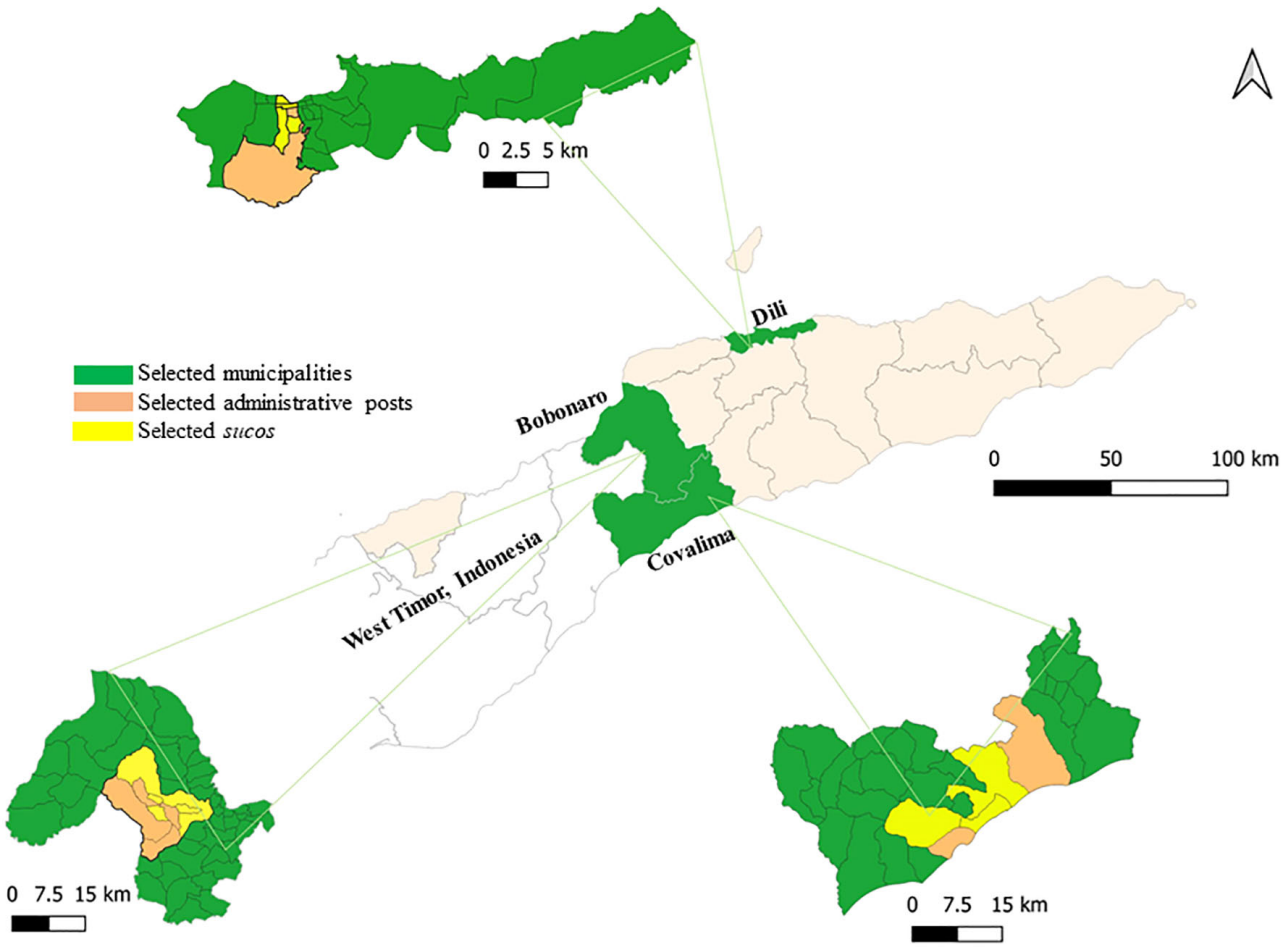
Map of Timor-Leste indicating the study locations. The survey was conducted in selected *sucos*, administrative posts within the three highlighted municipalities of Dili, Bobonaro, and Covalima.

This cross-sectional study was conducted from mid-February to mid-March 2023 in Timor Leste, a small country that constitutes the eastern half of Timor Island. It is administratively divided into municipalities, administrative posts, and *sucos*, which are the smallest administrative unit. The country has a population of 1.3 million people distributed across 14 municipalities and 250,270 private households (Timor Leste National Institute of Statistics, 2023). This study was conducted in three municipalities: Dili, Bobonaro, and Covalima (**Figure 1**). Dili is the capital city, has a total land area of 364 km^2^, and is the most populous municipality, with 324,738 inhabitants (Timor Leste National Institute of Statistics, 2023). Bobonaro is located in the northwest of Timor-Leste, and has a land area of 1,378 km^2^ (Bobonaro Municipality Authority, 2023) and a population of 106,639 people in 20,820 households distributed across 6 administrative posts and 50 *sucos* (Timor Leste National Institute of Statistics, 2023). Covalima lies in the southwest of Timor-Leste, and has a land area of 1,207 km^2^ (Covalima Municipality Authority, 2023) and a population of 73,933 people in 15,678 households distributed across 7 administrative posts and 30 *sucos* (Timor Leste National Institute of Statistics, 2023). Covalima and Bobonaro share land borders with the East Nusa Tenggara province of Indonesia.

### Sample size

**Figure 2:**
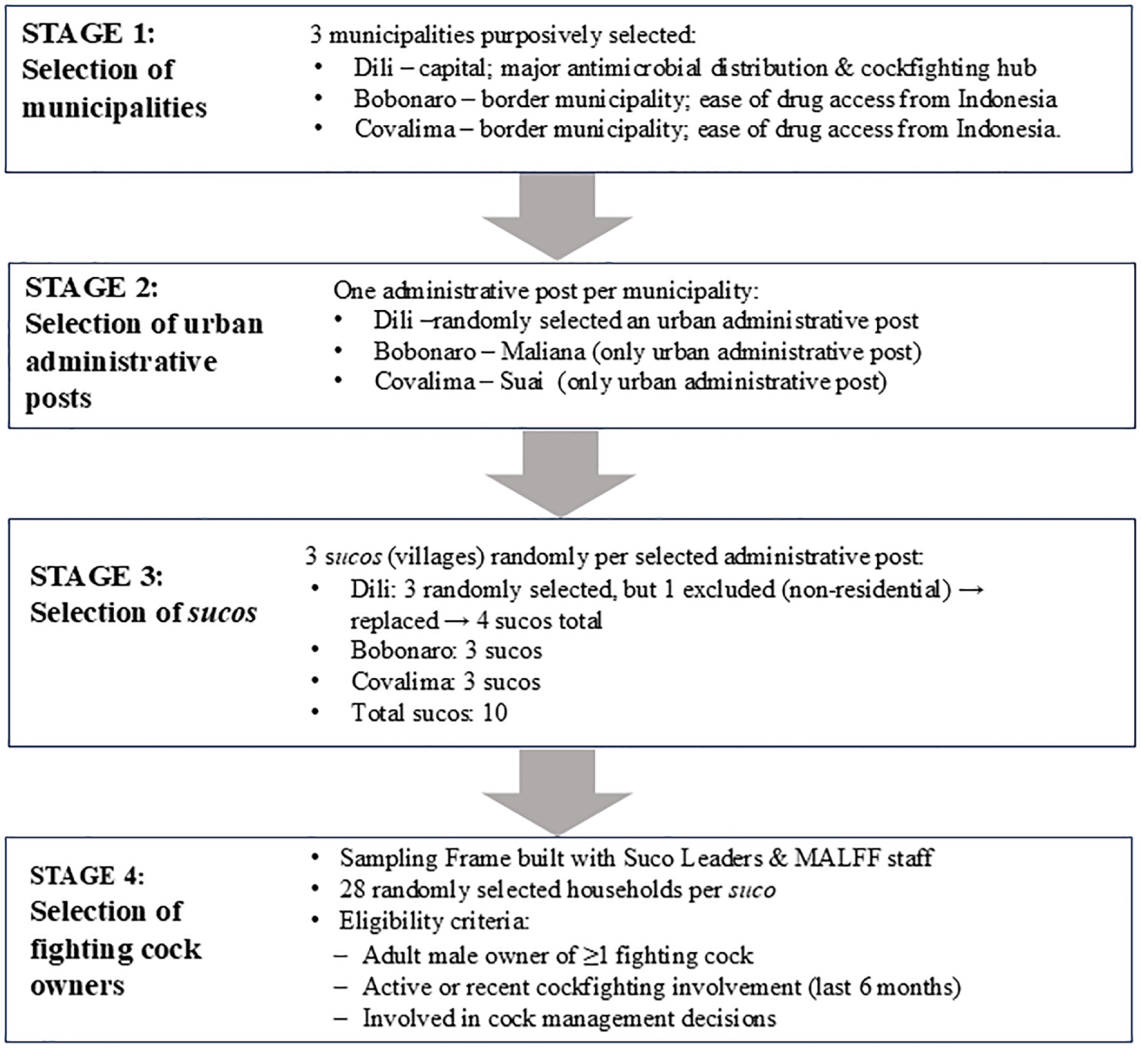
Flowchart of the multi-stage sampling design for the selection of fighting cock owners in Dili, Bobonaro, and Covalima municipalities.

A target sample size of 246 fighting cock owners was estimated using Statulator (Dhand and Khatkar, 2014), based on the following assumptions: 20% expected proportion of participants with knowledge about antimicrobials based on expert opinion; confidence level of 95%; and margin of error of 5%. Multi-stage sampling was employed to select study areas and study participants (**Figure 2**).

### Selection of study areas

The three municipalities of Dili, Bobonaro, and Covalima were purposively selected. Dili, was selected as it is a central point for antimicrobial distribution chains and also a key cockfighting hub, where cockfighting occurs daily (Hutt, 2015). The two border municipalities of Bobonaro and Covalima were selected because of the ease of access to antimicrobials from Indonesia. Oecusse, the third border municipality, was excluded because of its remote location. In each municipality, one urban administrative post was selected, as in urban locations it is easier to access antimicrobials and cockfights occur every week. The single urban administrative posts in Bobonaro and in Covalima were selected and one urban administrative post was randomly selected in Dili. Three *sucos* in each of these administrative posts were then randomly selected. The number of *sucos* selected was limited to three in each administrative post for logistical reasons. In Dili, three *sucos* were randomly selected at the study design stage, but it was found during data collection that one of the three *sucos* had a limited number of fighting cock owners as it was predominantly a non-residential area. Therefore, another *suco* was randomly selected, resulting in selection of a total of four *sucos* in Dili.

### Selection of study participants

The study population was fighting cock owners and the sampling unit was households owning fighting cocks. As there is no register of fighting cock owners, the sampling frame for this study was constructed in consultation with *suco* leaders and local animal health technicians of the Timorese Ministry of Agriculture, Livestock, Forestry, and Fisheries (MALFF). The inclusion criteria for participants were (1) an adult male who owns at least one fighting cock in a household; (2) who is currently involved in cockfighting or was involved in the last six months; and (3) who is involved in decisions about the management of fighting cocks. Simple random selection was used to select 28 fighting cock owners per *suco* and an invitation to participate in the study was issued via a communication letter sent to the *suco* authorities. When a selected participant was unwilling or unavailable for an interview, a neighboring fighting cock owner residing within a 500-metre radius and listed in the sampling frame was selected.

### Data collection

A structured questionnaire developed in Research Electronic Data Capture (REDcap) was used in this study. It was written in English and then translated into Tetun by two Timorese researchers involved in the study who were fluent in English and Tetun. The questionnaire was adapted for use with fighting cock owners from a study that investigated the knowledge and practices about antimicrobial use and AMR among smallholder pig farmers in Timor-Leste (Ting et al., 2022a). The data were collected using a structured questionnaire consisting of 31 questions. All the questions were closed-ended, but a free-text option was included to capture descriptive data, where applicable. The questionnaire was divided into four sections: (1) demographic information, as an initial section (five questions) to capture essential data on participants to contextualize findings, (2) fighting cock ownership and management (eight questions), (3) knowledge about antimicrobials and AMR (five questions, designed to capture the most critical aspects of knowledge relevant to antimicrobial use and misuse). The questions progressed from establishing basic awareness about antimicrobials and key public health concepts like stewardship and resistance, to testing for functional understanding of an antimicrobial’s mechanism of action, and finally to evaluating their comprehension of the public health impact of antimicrobial resistance; and (4) practices around antimicrobial use (13 questions), which gathered detailed information on the owner’s behaviors related to antimicrobial use. The demographic questions included age, education, municipality, administrative post, and *suco* of residence. Data on gender were not collected, as the fighting cock industry in Timor-Leste is dominated by men, and they were thus the focus of this study. The section on fighting cock ownership included questions about the breed of the birds, flock size, ownership of other poultry, sources of fighting cocks, housing, and feed. The knowledge section included questions on knowledge about antimicrobials, how antimicrobials work, AMR, impacts of AMR, and knowledge about antimicrobial stewardship. Antimicrobials were referred to as antibiotics in the survey as it was felt that participants would be more likely to be familiar with this term. The practice section included questions on common medicines used in fighting cocks, sources of medicines, antimicrobial usage, including types of antimicrobials, frequency and routes of administration, signs that prompt the use of antimicrobials, sources of advice on using antimicrobials, and adherence to label instructions. Photos of the common veterinary antimicrobials available in Timor-Leste (Ting et al., 2021) and commonly available human antimicrobials were used in the interview as a visual aid for the identification of antimicrobials given to fighting cocks. All questions were worded neutrally to avoid leading the participants. The questionnaire was piloted with six fighting cock owners in Liquica municipality and the findings were used to revise the questionnaire before commencing the survey. The pilot data were not included in the final analysis. The questionnaire was administered by face-to-face interviews conducted by five MALFF researchers. Prior to data collection, the interviewers were trained on the study objectives, sampling strategy, data collection protocol, and research ethics. The interviews were conducted in Tetun and had an average duration of 30 minutes each. Study participants were individually interviewed at their residence or place of work. Written consent was obtained from each participant before the interview commenced. Before obtaining their consent, interviewers explained the details of the study, including its objectives, to the study participants. Participants were assured of anonymity and confidentiality, and interviews were held in a private setting. At the conclusion of the interview, a small bag of chicken feed weighing 200 grams was given to the participant as an incentive to encourage participation.

### Data management and analysis

During the data collection period, data were uploaded daily to the REDcap server hosted by the University of Melbourne. Upon the conclusion of data collection, the data were exported into Microsoft Excel 2021 for data cleaning, verification, and preparation for analyses. Free-text responses to 11 semi-closed questions were arranged into categories created retrospectively. Statistical analyses were performed using Jamovi version 2.6.26 (The Jamovi Project, 2022).

Categorical variables were summarized using frequency tables. For continuous variables, the selection of appropriate measures of central tendency and dispersion was guided by the nature of their statistical distribution. To determine this, each continuous variable was first assessed for normality. For the continuous variables that were found to be normally or approximately normally distributed, the mean was reported as the measure of central tendency and the standard deviation (SD) are reported as the measure of dispersion. For variables with skewed distributions, the median and the interquartile range (IQR) are reported. The associations between demographic variables (age, municipality, education) and knowledge and practice variables (antimicrobials, antimicrobial use, adherence to label instructions) were explored using multivariable binary logistic regression. The model-building process was guided by an *a priori* approach where all three demographic predictors were included in each model. Each model was subject to a series of diagnostic checks. Multicollinearity was assessed using the variance inflation factor and found to be low. Diagnostics for influential points (via Cook’s distance) and outliers (via standardized residuals) confirmed model stability. Omnibus likelihood ratio tests were used to assess the overall model fit and the unique contribution of each individual predictor. Finally, goodness-of-fit of each model was formally assessed using the Hosmer-Lemeshow test, which indicated a good fit for all three models (all p-values > 0.05). The regression results were reported using adjusted odds ratios (aOR) with corresponding 95% confidence intervals (CI), with the p-value for significance set at <0.05. This study did not calculate composite knowledge or practice scores from the survey responses. Instead, key outcomes of interest were analyzed as individual categorical variables and the proportions for these outcomes were calculated. For the multivariable binary logistic regression analyses, two of the three outcome variables (knowledge of antimicrobial resistance and adherence to label instructions) were dichotomized using the same logic: ‘Yes’ responses were coded as 1 (representing the desired outcome), while ‘No’ and ‘Don’t Know’ responses were combined and coded as 0 (representing a lack of the desired outcome). Participants’ location of origin was dichotomized. Participants from the capital municipality of Dili were assigned to one group, while participants from Bobonaro and Covalima were combined into ‘other municipalities’.

### Ethics approval

Human ethics approval for the study was obtained through the University of Melbourne (approval number 2023-25219-36611-5).

## Results

### Demographic characteristics of fighting cock owners and fighting cocks

A total of 275 male individuals who owned fighting cocks were interviewed across nine *sucos* in the municipalities of Dili, Bobonaro, and Covalima. Of these, 270 participants were included in the analyses, with five participants excluded because of extensive gaps in the data collected from them. The demographic characteristics of fighting cock owners and elements of fighting cock management are presented in **Table 1**. The participants had a median age of 44 (range 20-83) years. Around one-sixth (45/270) had not attended school, while the majority had completed formal education, with most having completed high school (45.6%, 123/270) or university education (17.8%, 48/270). The majority (81.9%, 221/270) owned native breed cocks, with a median number of four birds per owner (range 1-91). Apart from fighting cocks, around half (52.3%, 138/264) of them owned other poultry. Overall, 86.0% (160/186) of participants (93.2% in Bobonaro, 77.6% in Covalima, 92.3% in Dili) took birds to cockfighting rings.

**Table 1:** Demographic characteristics of fighting cock owners and fighting cock management practices of surveyed owners in Timor-Leste.

Variable/category	Frequency	Percentage	95% CI
Municipality (n=270)			
Bobonaro	88	32.6%	27.3-38.4%
Covalima	86	31.9%	26.6-37.6%
Dili	96	35.6%	30.1-41.4%
Education (n=270)			
No School	45	16.7%	12.7-21.6%
Elementary	54	20.0%	15.7-25.2%
High School	123	45.6%	39.7-51.5%
University	48	17.8%	13.7-22.8%
Age (years) (n=269)			
20-30	48	17.8%	13.7-22.8%
31-40	69	25.6%	20.7-31.1%
41-50	62	23.0%	18.4-28.4%
51-60	61	22.6%	18.0-28.0%
>60	30	11.1%	78.8-15.5%
Breed of fighting cocks^a^ (n=270)			
Native	221	81.9%	76.8-86.0%
Mixed	109	40.4%	34.7-46.3%
Purebred	35	13.0%	9.5-17.6%
Unknown	2	0.7%	0.4-2.9%
Number of fighting cocks (n=270)			
1-5	183	67.8%	62.0-73.1%
6-10	60	22.2%	17.7-27.6%
>10	27	10.0%	6.9-14.2%
Own other poultry (n=264)			
Yes	138	52.3%	46.3-58.2%
No	126	47.7%	41.8-53.7%
Source of fighting cocks^a^ (n=270)			
Bought through friends	141	52.2%	46.3-58.1%
Bought in the market	103	38.2%	32.6-44.1%
Bred at home	100	37.0%	31.5-43.0%
Imported from Indonesia	19	7.0%	4.5-10.8%
As gift from relatives and friend	11	4.1%	2.2-7.3.0%
Bought at cockfighting pit	3	1.1%	0.2-3.4%
Keeping fighting cocks^a^ (n=270)			
Tethered with shelter	183	67.8%	62.0-73.1%
Housed all the time	71	26.3%	21.4-31.9%
Free-roaming all the time	37	13.7%	10.1-18.4%
Housed with some free roaming or tethered	33	12.2%	8.8-16.7%
Tethered without shelter	22	8.1%	5.4-12.1%
Type of feeds given to fighting cocks^a^ (n=270)			
Grain	249	92.2%	88.3-94.9%
Commercial feeds	114	42.2%	36.5-48.2%
Household scraps	50	18.5%	14.3-23.6%
Other^b^	8	3.0%	1.4-5.9%

a Multiple response variable.

b Other feeds included rice bran, dried cassava, and papaya.

Fighting cocks were most commonly purchased, from friends (52.2%, 141/270) and/or at markets (38.1%, 103/270), with a small number of participants directly importing birds from Indonesia (7.0%, 19/270). Some participants bred their own fighting cocks (37.0%, 100/270), with 53 of them reporting no cock purchases and 15 stating that birds were not raised for fighting.

Only 13.7% (37/270) of the participants kept fighting cocks free-roaming all the time. The majority fully confined their birds, by tethering them with shelter (67.8%, 183/270) or in housing (26.3%, 71/270), with 33 reporting that their birds were housed, with some tethered and some free-roaming. Five participants housed fighting cocks individually in wooden or bamboo coops with slatted flooring. The most common feeds for fighting cocks were grain (92.2%, 249/270) and commercial feeds (42.2%, 114/270), with 18.5% (50/270) of participants also reporting feeding household scraps to the birds.

### Knowledge about antimicrobials and AMR

Knowledge about antimicrobials and AMR is summarized in **Table 2**. Only 34.6% (93/269) of participants reported that they knew what antimicrobials were. The majority said they did not know (45%, 121/269) or were unsure (20.4%, 55/269). The respondents living in urban municipalities had about 2.4-times (95% CI [1.4-4.1], p-value = 0.002) higher odds of being aware compared with those in rural areas. Higher education was also associated with greater awareness (aOR = 2.3, 95% CI [1.3-4.2], p-value = 0.007), while age had no meaningful effect (aOR = 0.9, 95% CI [0.5-1.5], p-value = 0.186) (**Table 3**). Of those who reported knowledge about antimicrobials, only one participant stated that antimicrobials are used for killing or inhibiting bacteria, while the majority said that antimicrobials were used to reduce pain (68.8%, 64/93). Around a quarter (25.8%, 24/93) of the participants said that antimicrobials were used for killing or inhibiting viruses. A small proportion said that antimicrobials were used for treating fight wounds (9.7%, 9/93), treating and preventing diseases (9.7%, 9/93), reducing fever (4.3%, 4/93), increasing bird stamina (3.2%, 3/93), increasing the bird’s immunity (2.2%, 2/93), and killing or inhibiting parasites (1.1%, 1/93). When asked if they had heard of any antimicrobial stewardship awareness program, less than 5% (13/269) said they had heard of one. Those that had heard of an antimicrobial stewardship awareness program came across it through social media (4/13), human and animal health workers (4/13), and television (3/13).

**Table 2:** Knowledge about antimicrobials and antimicrobial resistance among fighting cock owners in Timor-Leste.

Variable/category	Frequency	Percentage	95% CI
Knowledge of antibiotics (n=269)			
Yes	93	34.6%	29.1-40.4%
No	121	45.0%	39.2-51.0%
Not sure	55	20.4%	16.1-25.7%
Do you know how antibiotics work?^a^ (n=93)			
Reduce pain	63	67.7%	57.7-76.4%
Kill/inhibit viruses	24	25.8%	18.0-35.6%
Treat fight wounds	9	9.7%	5.0-17.7%
Treat and prevent diseases	9	9.7%	5.0-17.7%
Reduce fever	4	4.3%	1.4-11.0%
Increase bird’s stamina	3	3.2%	0.8-9.6%
Increase bird’s immunity	2	2.2%	0.2-8.1%
Kill/inhibit bacteria	1	1.1%	0.0-0.6%
Kill/inhibit parasite	1	1.1%	0.0-0.6%
Have you heard of any antibiotic stewardship awareness program? (n=269)			
Yes	13	4.8 %	2.8-8.2%
No	188	69.9%	64.1-75.1%
Don’t know	68	25.3%	20.5-30.8%
Have you heard about antibiotic resistance? (n=267)			
Yes	16	6.0 %	3.7-9.6%
No	251	94.0%	90.4-96.3%
What do you think the impact of antibiotic resistance is?^a^ (n=16)			
Antibiotic is less effective	6	37.5%	18.5-61.5%
Antibiotic is more effective	7	43.8%	23.2-66.8%
Other^b^	4	25.0%	9.9-50.1%
Don’t know	2	12.5%	2.5-37.5%

a Multiple response variable.

b Others include low bird immunity, coughing, and overdosing.

**Table 3:** Reported practices on medicine and antibiotic use among fighting cock owners in Timor-Leste.

Characteristic	Category (n)	Awareness of antimicrobials N (%)	Awareness of antimicrobials aOR (95% CI)	Awareness of antimicrobials p-value	Use of antimicrobials N (%)	Use of antimicrobials aOR (95% CI)	Use of antimicrobials p-value	Adherence to label instructions N (%)	Adherence to label instructions aOR (95 % CI)	Adherence to label instructions p-value
Municipality	Dili (95)	47 (49.5)	2.4 (1.4-4.1)	0.002	73 (76.8)	0.9 (0.5-1.8)	0.928	32 (43.8)	0.3 (0.2-0.5)	0.847
	Other municipalities (174)	46 (26.4)	Ref		132 (75.9)	Ref		45 (34.1)	Ref	
Education Level	High school or higher (171)	72 (42.1)	2.3 (1.3-4.2)	0.007	131 (76.6)	1.0 (0.5-1.8)	0.907	60 (45.8)	2.5 (1.3-4.9)	0.008
	No education or elementary (98)	21 (21.4)	Ref		74 (75.5)	Ref		17 (22.9)	Ref	
Age Group	≤ 40 Years (116)	43 (37.1)	0.9 (0.5-1.5)	0.567	90 (77.6)	1.1 (0.6-2.1)	0.668	41 (45.6)	1.5 (0.8-2.7)	0.186
	> 40 years (152)	50 (32.9)	Ref		114 (75.0)	Ref		36 (31.6)	Ref	

Very few participants (5.9%, 16/267) had heard about AMR. Of these, only six (37.5%) said it resulted in antimicrobials being less effective. The remainder said that AMR makes antimicrobials more effective (43.8%, 7/16) or were unsure about its impacts (12.5%, 2/16), while four participants stated that AMR reduces the immunity of birds, and causes coughing and abnormal respiration.

### Practices associated with use of medicines

A total of 200 participants (74.9%, 200/267) gave medicines to their fighting cocks (**Table 4**), with the most frequently reported medicines being antibiotics (93%, 186/200), multivitamins (44%, 88/200), and performance enhancers, commonly referred to as ‘doping’ (20.5%, 41/200). A few participants reported the use of pain relievers (4.5%), antiparasitic drugs (1.5%), or local remedies (papaya leaves, fried oil, local alcoholic drinks, coffee powder) in fighting cocks. Four participants mentioned other medicines, including mineral supplements and anti-inflammatory drugs. Medicines used in fighting cocks were obtained from various sources, with the most common being agriculture shops (47.5%, 95/200), pharmacies (38.5%, 77/200), and markets (26.5%, 53/200) (**Table 4**). Other sources of medicines used in fighting cocks included left-over human medicines (9.5%, 19/200), kiosks (4.0%, 8/200), veterinary clinics (2.5%, 5/200), importation from overseas (2.5%, 5/200), through friends (1%, 2/200), and left-over animal medicines (0.5%, 1/200).

**Table 4:** Practices related to antimicrobial use among fighting cock owners in Timor-Leste.

Variable/category	Frequency	Percentage	95% CI
Do you or does anyone else give any medicine to your fighting cocks? (n=267)			
Yes	200	74.9 %	69.4-79.7%
No	61	22.8 %	18.2-28.3%
Don’t know	6	2.3 %	0.9-5.0%
What medicine do you or does anyone else give to your fighting cocks?^a^ (n=200)			
Antibiotics	186	93.0%	88.5-95.9%
Multivitamins	88	44.0%	37.3-50.9%
‘Doping’/performance enhancer	41	20.5%	15.5-26.7%
Pain reliever	9	4.5%	2.3-8.5%
Antiparasitic	3	1.5%	0.3-4.6%
Local remedies^b^	4	2.0%	0.6-5.3%
Others^c^	4	2.0%	0.6-5.3%
Source of medicines^a^ (n=200)			
Agriculture shop	95	47.5%	40.7-54.4%
Pharmacy	77	38.5%	32.0-45.4%
Market	53	26.5%	20.9-33.1%
Left-over at home from treating a person	19	9.5%	6.1-14.5%
Kiosk	8	4.0%	1.9-7.9%
Veterinary clinic	5	2.5%	0.9-5.9%
Self-import	5	2.5%	0.9-5.9%
Friend	2	1.0%	0.6-3.9%
Left-over at home from treating other animals	1	0.5%	0.0-3.1%
Do you or does anyone else give your fighting cocks antibiotics? (n=269)			
Yes	205	76.2 %	70.7-80.9%
No	64	23.8 %	19.1-29.3%
Which antibiotics do you commonly use?^a^ (n=205)			
Human antibiotics			
Amoxicillin trihydrate (*Amoxicillin*)	159	77.6%	71.3-82.7%
Ampicillin	111	54.2%	47.3-60.8%
Tetracycline (*Supertetra*)	47	22.9%	17.7-29.2%
Veterinary antibiotics			
Tetracycline and erythromycin (*Tetrachlor)*	48	23.4%	18.1-29.7%
Trimethoprim and sulfadiazine (*Trimezyn*)	29	14.2%	10.0-19.7%
Oxytetracycline (*Medoxy LA*)	20	9.8%	6.4-14.7%
Amoxicillin and colistin sulphate (*Amoxitin*)	13	6.3%	3.7-10.7%
Penicillin and streptomycin (*Penstrep*)	2	0.9%	0.5-3.8%
Don’t know	3	1.5%	0.3-4.5%
Other^d^	33	16.1%	11.7-21.8%
What signs in animals will prompt you to use antibiotics?^a^ (n=205)			
Fight wounds	175	85.4%	79.8-89.6%
Respiratory signs	54	26.3%	20.8-32.8%
Diarrhoea	43	21.0%	16.0-27.1%
Skin infection	40	19.5%	14.7-25.5%
Fever	10	4.9%	2.6-8.9%
Listlessness	11	5.4%	3.0-9.5%
Use for disease prevention	4	1.9%	0.7-5.5%
To increase bird stamina	3	1.5%	0.3-4.5%
Others^e^	7	3.4%	1.5-7.1%
How often do you or does anyone else give your fighting cocks antibiotics? (n=204)			
When needed	73	35.8%	29.5-42.6%
A day after the fight	59	28.9%	23.1-35.5%
Weekly	56	27.5%	21.8-34.0%
Monthly	7	3.4%	1.6-7.1%
A day before the fight	9	4.4%	2.3-8.3%
How do you or does anyone else administer antibiotics to your fighting cocks? (n=205)			
Oral	181	88.3%	83.1-92.0%
Applied directly to the fight wounds	49	23.9%	18.6-30.2%
Injection	31	15.1%	10.9-20.7%
In water	12	5.9%	3.3-10.1%
In feed	3	1.5%	0.3-4.5%
Others^f^	5	2.4%	0.9-5.8%
Where do you get advice about using antibiotics?^a^ (n=205)			
Friends	113	55.1%	48.3-61.8%
Family	42	20.5%	15.5-26.6%
Neighbours	14	6.8%	4.1-11.3%
Online videos	11	5.4%	3.0-9.5%
Agriculture shops	5	2.4%	0.9-5.8%
Veterinarian	2	1.0%	0.6-3.8%
Veterinary paraprofessional	8	3.9%	1.9-7.7%
Pharmacy	2	0.9%	0.6-3.8%
Extension worker	1	0.5%	0.0-3.1%
No advice	72	35.1%	28.9-41.9%
Do you follow label instructions when using antibiotics? (n=205)			
Yes	77	37.6%	31.2-44.4%
No	110	53.7%	46.8-60.3%
Don’t know	18	8.8%	5.6-13.6%
If you don’t use antibiotics, why? (n=64)			
Lack of knowledge about antibiotics and how antibiotics work	21	31.3%	21.9-43.9%
Not available nearby	17	26.6%	17.3-38.6%
Antibiotics are ineffective	8	12.5%	6.3-23.1%
Preference for local remedies	8	12.5%	6.3-23.1%
Birds are not sick	7	10.9%	5.2-21.3%
Antibiotics are expensive	6	9.4%	4.1-19.4%
Do not want to use	3	4.7%	1.2-13.6%
“Because we cannot eat the dead bird if given antibiotics”	1	1.6%	0.0-9.3%

a Multiple-response variable.

b Local remedies included papaya leaves, fried oil, local alcoholic drinks, and coffee powder.

c Others included mineral supplements and anti-inflammatory drugs.

d Others: 33 of the study participants mentioned others, but only four people correctly identified three antibiotics, doxycycline (*Doxyvet*), and oxytetracycline HCl and erythromycin thiocyanate (*Tetrafein*). Twenty-nine wrongly identified acetaminophen (*Paracetamol*), chlorpheniramine maleate, phenylpropanolamine hydrochloride (*Mixagrip*), ibuprofen, or performance enhancers as antibiotics.

e Others include darken comb, swollen eyes, inappetence, and simply stating sick bird.

f Others included pounding the antibiotic tablet and applying into the wound as a powder or spray.

### Practices associated with antimicrobial use

When asked specifically about antibiotics, 76.2% (205/269) said antibiotics had been given to their fighting cocks. Human antimicrobials were given by 89.8% (184/205) of these participants their fighting cocks, with the most common drugs given being amoxicillin trihydrate (77.5%, 159/205), ampicillin (54.2%, 111/205) and tetracycline (22.9%, 47/205). Veterinary antimicrobials were given to fighting cocks by 37.1% (76/205) of these participants, with the most common being tetracycline and erythromycin (*Tetrachlor)* (23.4%, 48/205), trimethoprim and sulfadiazine (*Trimezyin*) (14.2%), oxytetracycline (*Medoxy LA*) (9.8%), amoxicillin and colistin sulphate (*Amoxitin*) (6.3%), and penicillin and streptomycin (*Penstrep*) (0.9%).

When asked whether any other antibiotics were given to their fighting cocks, around 16% (33/205) provided a range of responses, but only four participants correctly named additional antimicrobials, doxycycline (*Doxyvet)*, and oxytetracycline HCl and erythromycin *(Tetrafein*). The majority of them (17/33) named other classes of drugs, including human analgesics (paracetamol - 17/33; ibuprofen - 3/33), performance enhancers (5/33), dypirone (*Sulpidon*) (2/33), and povidone-iodine (*Betadine*) (2/33).

**Figure 3:**
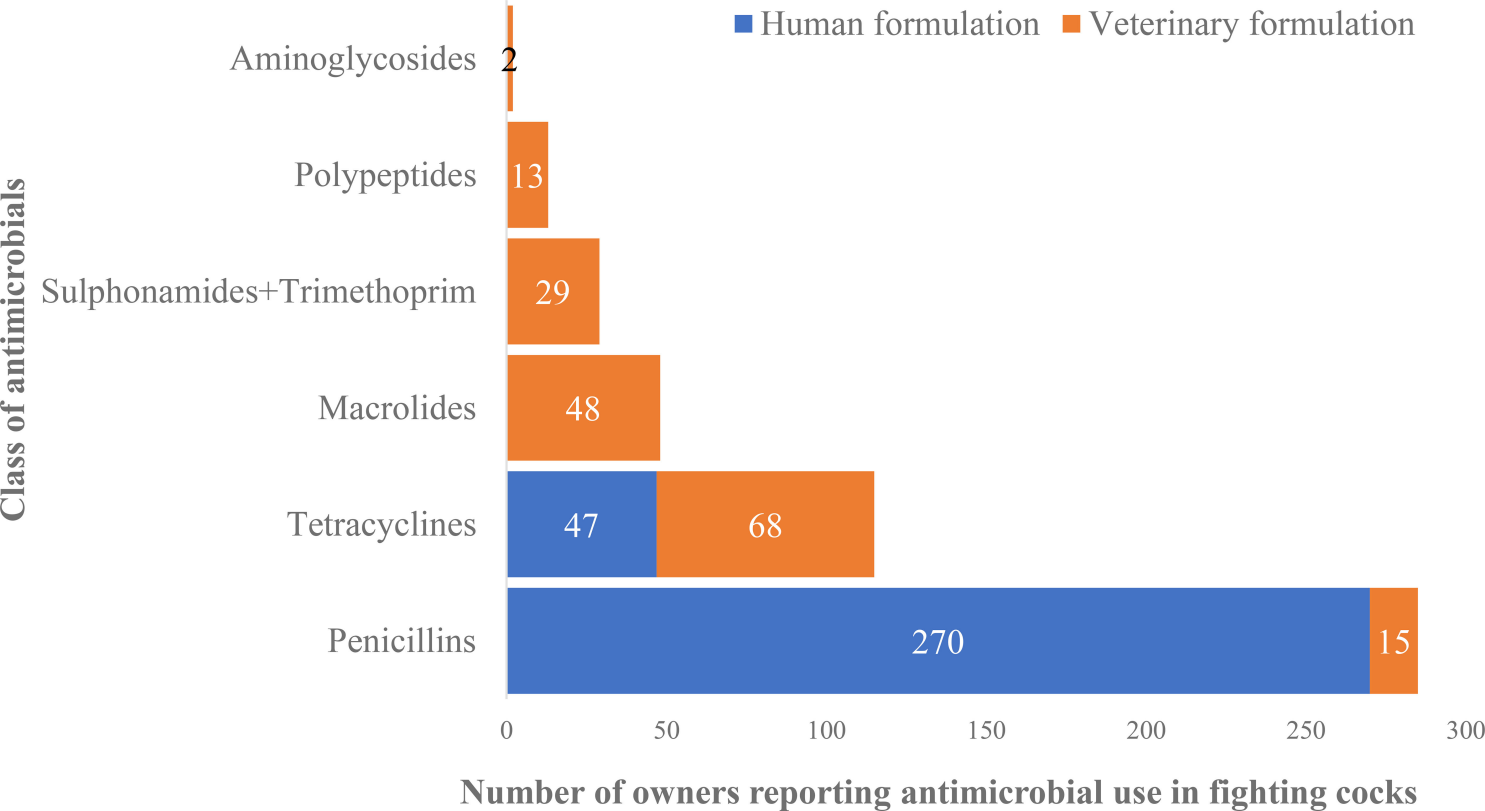
Reported use of antimicrobials by class and formulation among fighting cock owners in Timor-Leste. Three of the products included active ingredients belonging to more than one class.

The classifications of the antimicrobials given by participants to their fighting cocks, by class and formulation (veterinary versus human) are shown in **Figure 3**. Six antimicrobial classes were commonly given to fighting cocks, with the most frequently reported being penicillins, tetracyclines, and macrolides. Human formulations constituted the majority (94.7%) of the penicillins given to fighting cocks, while both human(40.9%) and veterinary (59.1%) formulations of tetracyclines were administered to birds, with only veterinary formulations of the less commonly administered classes given to fighting cocks.

The most common reason for the use of antimicrobials was to treat fight wounds (85.4%, 175/205). This was followed by respiratory signs (26.3%, 54/205), diarrhea (21.0%, 43/205), skin infections (19.5%, 40/205), listlessness (5.4%, 11/205), and fever (4.9%, 10/205). Some participants reported prophylactic use to prevent disease (4/205) or to increase bird stamina and strength (2/205), including one owner who administered antimicrobials when the bird was tired after the fight. Seven participants mentioned other clinical manifestations that prompted the use of antimicrobials, including darkened combs (2/7), ‘sick bird’ (2/7), swollen eyes (1/7), and inappetence (1/7).

Around 30% of participants had a standard schedule for using antimicrobials in fighting cocks, regardless of fighting activity, using them every week (56/204) or every month (7/204). Around a third used antimicrobials based on fighting activity, either a day after the fight (73/204) or before the fight (9/205). The remaining participants (73/204) said that antimicrobials were administered when needed, for example, when a bird was sick (47/73) or wounded (10/73). The most common route of administration was oral, as tablets (88.3%, 181/205), followed by direct application to the fight wounds (23.9%), and injection (15.1%). Fewer than 10% (15/205) administered antimicrobials in feed or water.

Advice on antimicrobial use in fighting cocks was not sought by 35.1% participants. For those who sought advice, the predominant sources were friends (55.1%, 113/205) and family (42/205), with only 5% (10/205) seeking professional advice from veterinarians or veterinary paraprofessionals. Other sources of advice included online videos to observe how others used antimicrobials (11/205), pharmacies (2/205), and extension workers (1/205). More than half (53.7%, 110/205) did not adhere to label instructions when using antimicrobials and a further 8.8% (18/205) were unsure of what a label instruction was.

The reasons 23.8% (64/269) of participants did not use antimicrobials in their fighting cocks were varied and included lack of access due to their distant location (26.6%, 17/64), the high price of antimicrobials (9.4%, 6/64), their belief that antimicrobials were ineffective (12.5%, 8/64), their lack of knowledge about antimicrobials and how they work (20/64), their preference for local remedies (8/64), because their birds were not sick (7/64), and their reluctance to use antimicrobials (3/64), with one person saying “because we cannot eat the dead bird if given antibiotics”.

There was no evidence of an association between the use of antimicrobials and municipality (aOR = 0.9, 95% CI [0.5-1.8], p-value = 0.928), education (aOR = 1.0, 95% CI [0.5-1.8], p-value = 0.907) or age (aOR = 1.1, 95% CI [0.6-2.1], p-value = 0.668). However, education was associated with adherence to label instructions when using antimicrobials (aOR = 2.5, 95% CI [1.3-4.9], p-value = 0.008) (**Table 3**).

## Discussion

As a significant global challenge, AMR is partly driven by the use of antimicrobials in animals. Therefore, understanding about antimicrobial use in fighting cocks is a component of the knowledge needed to inform efforts to mitigate the development of resistant pathogens and their adverse impact on both animal and human health. To our knowledge, this is the first study that has examined knowledge about, and practices associated with, antimicrobial use in fighting cocks in Timor-Leste and the Pacific. Management of fighting cocks is more intensive than that of backyard local chickens in Timor Leste, as the majority of the fighting cocks are tethered with shelter (67.8%) and are fed predominantly with grains (92.2%) and commercial feeds (42.2%). In contrast, local backyard chickens in Timor-Leste are raised in small-scale flocks that are mostly free-roaming and scavenge their own food (Jong, 2016).

Knowledge among fighting cock owners in Timor-Leste about antimicrobials was poor, with only 34.6% of participants knowing what antimicrobials were, and only one participant able to correctly indicate how they worked. Our study indicates that two key socio-demographic factors — geographic location and educational attainment are strong predictors of awareness. Urban participants had 2.4-times higher odds of awareness, probably because they have greater access to diverse information channels, including mainstream media, digital platforms, and public awareness campaigns, which are often concentrated in cities. Similarly, the strong association between higher education and greater awareness (aOR = 2.3, 95% CI [1.3-4.2], p-value = 0.007) aligns with established research on antimicrobials and AMR (Simegn and Moges, 2022). The predominant misconceptions were that they reduced pain (68.8%), killed or inhibited viruses (25.8%), or reduced fever (22.6%). Similar misconceptions were also noted among government animal health workers in Timor-Leste (Ting et al., 2022b). Three quarters of the owners surveyed used medicines in their fighting cocks, and the majority of these (93.0%, 186/200) included antimicrobials among the medicines that they used. However, when a specific question about antibiotic use was asked, 76.2% (205/269) said they used antibiotics. This discrepancy in the number of owners reporting antimicrobial use might have been because some were unable to distinguish between antimicrobials and other medicines, as some owners misclassified other medicines as antimicrobials. This gap in knowledge about antimicrobials is likely to contribute to inappropriate usage. Similar findings were reported in a study that investigated the knowledge and practices associated with antimicrobial use in pig farmers in Timor-Leste (Ting et al., 2022a). We have not identified comparable studies in fighting cock owners in other countries, but the lack of knowledge about antimicrobials revealed here is consistent with findings from studies of small-scale livestock and poultry farmers in other LMICs (Moffo et al., 2020; Benavides et al., 2021). Higher levels of knowledge about antimicrobials and AMR have been found in studies of ruminant owners in Malaysia (Sadiq et al., 2018), poultry farmers in Vietnam (ThiHuong-Anh et al., 2020), and cattle owners in India (Dhayal et al., 2023). The study described here found that only 6% of fighting cock owners had heard of AMR. This is a very low level of knowledge and may be due to the lack of awareness campaigns in Timor-Leste, as fewer than 5% of respondents had heard of awareness programs about antimicrobial stewardship. Limited awareness about antimicrobial stewardship has also been noted in a previous study in Timor-Leste (Ting et al., 2022a). Based on the few studies that have been conducted in the broader Asia-Pacific region, farmer knowledge about AMR is variable, with limited knowledge reported in Fiji (Shah et al., 2023), but notably higher levels of knowledge among backyard and small commercial poultry farmers in Nepal (Subedi et al., 2023). The lack of knowledge about antimicrobials and AMR, even among educated owners, demonstrates an urgent need for awareness campaigns in Timor-Leste. Despite the national-level education campaigns that have been carried out by the Ministry of Health, MALFF, and their development partners (WHO, 2022), targeted campaigns are needed to raise awareness about AMR among sub-groups in the general population that are involved in the use of antimicrobials, such as fighting cock owners. The educational messages need to be crafted to address key misconceptions identified in this study and in a previous study of smallholder pig owners in Timor-Leste (Ting et al., 2022a).

Our study has shown that the use of antimicrobials in fighting cocks is high (76.2%). Although the proportion of participants reporting use in fighting cocks in Timor-Leste was slightly lower than the proportion of farmers reporting use in poultry in Nepal (Subedi et al., 2023), it was more than 20 times higher than the proportion of pig farmers in Timor-Leste who reported using antimicrobials in their animals (Ting et al., 2022a). The sources most commonly used by fighting cock owners to obtain medicines, including antimicrobials, were agriculture shops (47.5%), as was the case for pig farmers in Timor-Leste (Ting et al., 2022a), and other livestock keepers in LMICs (Barroga et al., 2020; Benavideset al., 2021). Other sources of antimicrobials used by fighting cock owners included pharmacies (38.5%), markets (26.5%), and kiosks (4.0%), which are very small grocery shops in Timorese communities (Mau-Quei and Cameron, 2019). Most of these sources were also found to be sources of antimicrobials used by government animal health workers in Timor-Leste (Ting et al., 2022b). Thus, these sources could be targeted as potential intervention points for raising awareness, promoting prudent antimicrobial use, and regulating antimicrobial access, important areas for intervention identified in the Timor-Leste National Action Plan for AMR (WHO, 2022).

The antimicrobials most commonly used in fighting cocks were formulations for humans containing actives in the penicillin and tetracycline classes. The use of antimicrobials intended for human use in animals was also reported previously by government animal health workers, who also reported the use of antimicrobials in fighting cocks (Ting et al., 2022b). The use of human formulations of penicillins (amoxicillin and penicillin) and tetracyclines in domestic animals has also been reported in other LMICs, such as Ethiopia (Geta and Kibret, 2021), Tanzania, India, and Uganda (Myers et al., 2022). The widespread use of human formulations in fighting cocks reflects the ease of access to medical antimicrobials in Timor-Leste, and is clear evidence of misuse that needs to be addressed. The most commonly used veterinary formulations contained actives in the tetracycline and macrolide classes. This differs from the findings of studies of poultry farmers in Nepal, where tetracyclines, aminoglycosides, and fluoroquinolones were the antimicrobials most commonly used (Subedi et al., 2023), but is similar to the findings from a study in Thailand, which found that tetracyclines and macrolides were the antimicrobials most commonly used in livestock (Lekagul et al., 2023).

Nearly all of the administration of antimicrobials to fighting cocks is done without professional guidance from veterinarians or veterinary paraprofessionals, as has been seen in studies of livestock owners in other LMICs (Ojo et al., 2016). This may be because of the inaccessibility of veterinary services or a lack of veterinary professionals or paraprofessionals, as has been reported in other studies in Timor-Leste (De Almeida et al., 2021; Hunter et al., 2022; Ting et al., 2022b). This could explain why the majority (82.4%) of the participants sought advice from friends, family members, and neighbors. The label is an alternative source of information to guide appropriate use of antimicrobials, but this study highlighted that more than half (53.7%) of the owners who administered antimicrobials to their birds did not adhere to label instructions. This would be understandable for those (45/270) of the bird owners who had had no education, but lack of adherence to the label instructions among educated owners is concerning. More than a quarter (27.5%) of the participants reported monthly administration of antimicrobials to fighting cocks and 3.4% administered them to their birds on weekly basis. Such practices are likely to select for AMR and may be attributable to misconceptions that antimicrobials can improve the stamina and bravery of the birds, key qualities for cock fighting (Kavesh, 2021). Some fighting cock owners (9.5%) reported using leftover human antimicrobials in fighting cocks, a practice also reported by owners of livestock in other LMICs, such as Fiji (Shah et al., 2023) and India (Dhayal et al., 2023). Although the proportion of participants engaging in this practice was low, it is concerning practice requires intervention to reduce the risk that it may contribute to the emergence and spread of resistance, particularly to higher importance antimicrobials. The findings of this study showing widespread antimicrobial use coupled with poor knowledge are symptomatic of a larger systemic issue: the lack of a national regulatory framework governing the use of antimicrobials in Timor-Leste (WHO, 2022). This allows for unrestricted, over-the-counter access to these medicines, which facilitates the misuse we documented in this study and probably encourages similar behavior across the broader livestock sector.

A limitation of this study is its focus on fighting cock owners’ practices without concurrent collection of samples from fighting cocks to determine the prevalence of AMR. Thus, our study documents the high-risk behaviors for AMR selection, but cannot draw conclusions about the current AMR landscape in the fighting cock population. This is an important gap, given that a recent study in Timor-Leste, although limited in its sample size (n=72), did find evidence of resistance in *E.coli* to ampicillin and tetracyclines in fighting cocks (Pereira et al., 2024). Building on our findings, future studies should focus on collecting samples directly from birds on farms and at cockfighting pits, with the permission of fighting cock owners, which would enhance our understanding about the associations between antimicrobial use and AMR in fighting cocks. Given the high and inappropriate use of antimicrobials in fighting cocks, these birds should be targeted for ongoing surveillance for antimicrobial resistance and residues. The public health impacts of antimicrobial residues are variable, but a key concern is the apparent link between rising AMR and allergic reactions (Hassan et al., 2021; Arsène et al., 2022). Monitoring of antimicrobial residues in the meat of fighting cocks may help to generate valuable information to inform strategies for intervention.

Interviews were only conducted with males because of the well-documented predominance of men in the cockfighting industry in Timor-Leste (Hicks, 2006; Hutt, 2015; Wu, 2022; De Andrade, 2023), Indonesia (Sanjatmiko, 2021), and in other LMICs (Kalof, 2014). However, we acknowledge that this gender restriction was a study limitation and recommend that future research should investigate the role of women in the cockfighting industry, especially as we found that some owners specialized in breeding fighting cocks and did directly participating in cock fights, as typically women in Timorese households are responsible for raising and selling other poultry (Wong, 2017). Moreover, gender may also influence access to information and decision-making about antimicrobial use. We did not attempt to investigate dose rates, duration of use or knowledge about withdrawal periods. Lack of adherence to withdrawal periods is the major reason antimicrobial residues are found in food of animal origin (Bacanlı and Başaran, 2019; Thi Huong-Anh et al., 2020). More than half (52.3%) of the respondents indicated that they also had other poultry, which are likely to kept in the same environment. This may be a risk for horizontal transmission of AMR bacteria between fighting cocks and other poultry and other animals.

A further limitation was our study’s sample size. While the sample size was calculated using an expected knowledge proportion of 20% based on preliminary local expert consultation, using a more conservative 50% proportion, a standard approach when no prior knowledge is available, would have led to a larger and more robust sample size. The decision to proceed with the smaller sample size was necessitated by significant logistical and resource constraints. While this cross-sectional study provides a snapshot in time about our understanding of awareness and practices of antimicrobial use among fighting cock owners, future research aimed at having more robust quantitative designs to understand how knowledge and use patterns evolve over time are essential. Our findings may not be generalizable to all poultry farmers in Timor-Leste, as we deliberately focused on the owners of fighting cocks. This allowed us to conduct an in-depth analysis of this specific high-risk group. However, future comparative research is indeed warranted. A study comparing the knowledge, attitudes, and practices of fighting cock owners with those of farmers raising poultry for meat or eggs would provide a more comprehensive understanding of the varying drivers of antimicrobial use and AMR risk across the poultry sectors in Timor-Leste.

## Conclusion

This study found that fighting cocks are frequently given antimicrobials by owners in Timor-Leste and that these owners lack a fundamental understanding about these medicines and the threat of AMR. This uninformed use creates a high-risk environment for the emergence and spread of resistant pathogens in fighting cock industry settings, posing a potential threat to both animal and human health. Therefore, a multi-pronged intervention strategy is urgently required. This must include strengthening regulations to control antimicrobial access and use, establishing AMR surveillance, and developing educational campaigns tailored for low-literacy owners through existing animal health extension services. Such interventions are essential to mitigate the public health risks of AMR in Timor-Leste.

## Data Availability

The raw data supporting the conclusions of this article will be made available by the authors, without undue reservation.
